# Preventing Zika Virus Infection during Pregnancy Using a Seasonal Window of Opportunity for Conception

**DOI:** 10.1371/journal.pbio.1002520

**Published:** 2016-07-28

**Authors:** Micaela Elvira Martinez

**Affiliations:** 1 Department of Ecology and Evolutionary Biology, Princeton University, Princeton, New Jersey, United States of America; 2 Global Health Program, Woodrow Wilson School of Public and International Affairs, Princeton University, Princeton, New Jersey, United States of America; The Pennsylvania State University, UNITED STATES

## Abstract

It has come to light that Zika virus (ZIKV) infection during pregnancy can result in trans-placental transmission to the fetus along with fetal death, congenital microcephaly, and/or Central Nervous System (CNS) malformations. There are projected to be >9,200,000 births annually in countries with ongoing ZIKV transmission. In response to the ZIKV threat, the World Health Organization (WHO) is strategically targeting prevention of infection in pregnant women and funding contraception in epidemic regions. I propose that the damaging effects of ZIKV can be reduced using a seasonal window of opportunity for conception that may minimize maternal exposure. Like other acute viral infections—including the related *flavivirus*, dengue virus (DENV)—the transmission of ZIKV is anticipated to be seasonal. By seasonally planning pregnancy, this aspect of pathogen ecology can be leveraged to align sensitive periods of gestation with the low-transmission season.

## Abstract 2

Zika infection during pregnancy can result in microcephaly, fetal malformations, and miscarriage. A window of opportunity exists to reduce this risk by planning conception seasonally to align sensitive periods of gestation with the low-transmission season.

## Author Summary

Scientific consensus has now been reached that intrauterine Zika virus (ZIKV) infection can result in infection of the fetus and subsequent fetal death, congenital microcephaly, and/or Central Nervous System (CNS) malformations. Preliminary data suggest miscarriage and congenital Zika syndrome (CZS) are most likely when maternal infection occurs early in pregnancy, but fetal abnormalities have been found in women infected with ZIKV during all three trimesters, indicating all trimesters are vulnerable. Like related *flavivirus* infections, ZIKV transmission is likely to be seasonal. I propose that the risk of ZIKV infection to pregnant women can be reduced using a window of opportunity for conception that will align sensitive periods of gestation with the low-transmission season for ZIKV.

## Zika Virus and Microcephaly

The Zika virus (ZIKV) is a mosquito-transmitted virus—vectored by *Aedes aegypti*—spreading rapidly across the globe [1]. Pregnant women infected with ZIKV risk severe fetal outcomes, including brain abnormalities—believed to be due to disruption of brain development caused by intrauterine infection—and death [2,3]. ZIKV was the suspected cause of the 2015/2016 outbreak of microcephaly in Brazil [4], and scientific consensus has now been reached that prenatal ZIKV infection causes microcephaly and other forms of brain abnormalities. Upon maternal infection, however, the risk of such fetal outcomes remains unknown [5]. In April 2016, the causal link between ZIKV and microcephaly was inferred via several independent lines of evidence, including (1) microcephaly and brain abnormalities in infants born to mothers with suspected or confirmed ZIKV infection during the first or second trimester of pregnancy, (2) the rare form of microcephaly in infants with congenital Zika syndrome (CZS), distinguishing it from microcephaly resulting from other causes, and (3) birth defects occurring in women with travel-acquired ZIKV, coupled with the low probability that these events were coincident and not causal [5]. Several reports from February to May 2016 have now provided strong evidence for the causal link. A retrospective study of the 2013/2014 ZIKV outbreak in French Polynesia found a 14-fold increase in severe microcephaly in newborns and fetuses following the epidemic; amniotic fluid tested positive for ZIKV in 4 of 7 women sampled after identification of fetal abnormalities [6]. In addition, in Brazil, 42 ZIKV-positive pregnant women were tested for fetal abnormalities. Adverse findings—including fetal death, microcephaly, and central nervous system (CNS) damage—were observed in 12 of the women. There were no abnormalities in ZIKV-negative women [2]. Lastly, the complete ZIKV genome was recovered from the brain of a fetus with microcephaly aborted by an expectant mother infected during the 13th week of gestation [7], and the Centers for Disease Control (CDC) reported on two newborns from Brazil with microcephaly who died shortly after birth, as well as two miscarriages; all tested positive for ZIKV [8]. In addition to the epidemiological evidence, newly developed mouse models of ZIKV have demonstrated that ZIKV strains from French Polynesia and Brazil can infect the fetus via the placenta and cause intrauterine growth restrictions and/or fetal loss [9,10]. Culture models of early brain development have also shown ZIKV can cause neural cell death [10,11].

Recognizing the incomplete picture of ZIKV in utero pathology, in February 2016, the WHO declared the cluster of microcephaly in Brazil to be a Public Health Emergency of International Concern, and the International Health Regulations Emergency Committee issued recommendations to reduce ZIKV infections in pregnant women [4]. In the United States, $1.9 billion has been requested of congress to respond to ZIKV domestically and internationally [12]. Maternally transmitted viral infections, such as ZIKV, can be prevented by protecting pregnant women from infection, but it is likely to be many years before a ZIKV vaccine or treatment is developed. Alternative preventative measures are therefore needed to protect women and their children from this emerging pathogen.

Two key components of the ZIKV response by governments and health agencies are (1) vector control and (2) preventing infection in pregnant women. The WHO’s ZIKV operational response plan includes control of *Aedes aegypti* mosquitoes and financing contraceptive services in affected areas to manage pregnancy and mitigate the impact of ZIKV [4]. At the CDC’s April 2016 Zika Action Plan Summit, the CDC Director acknowledged “the control of *Aedes aegypti* is challenging” and declared that decreasing the risk of ZIKV to pregnant women and women of childbearing age is a key priority [13].

Government officials in El Salvador, Colombia, and Ecuador have recommended women delay pregnancy while uncertainty surrounding ZIKV remains. The WHO ZIKV Q&A website (updated regularly) states, “Women wanting to postpone pregnancy should have access to a comprehensive range of reversible, long- or short-acting contraceptive options to the full extent of the law” [14]. The CDC has issued recommendations for ZIKV-exposed couples to delay pregnancy. Exposed women and exposed asymptomatic men are recommended to wait 8 weeks, and men with symptoms are recommended to wait 6 months [15]. No official stance on delaying pregnancy has been taken for unexposed women. Problematically, the public is receiving a mixed message highlighted by media coverage [16–18]. Given that extended delays of pregnancy may not be a viable option for millions of women living in ZIKV-epidemic regions, I propose a strategy that will reduce intrauterine ZIKV infection risk without requiring long-term delays of pregnancy. Specifically, I recommend that public health and research communities focus on three current ZIKV knowledge gaps:

1. seasonality of ZIKV transmission,
2. intrauterine transmission and pathology, and
3. immunity.

These aspects of ZIKV biology can be integrated with incidence data and mathematical models to inform interventions, including reducing transmission (i.e., vector-to-human and sexual) via vector control and behavioral changes, planning pregnancy to avoid the high-transmission season, launching vaccines once developed, and reducing intrauterine transmission and pathology. Knowledge gap 3 (immunity) will be particularly important for understanding the recurrent epidemic dynamics of ZIKV and CZS. If ZIKV antibodies either wane or do not fully protect from infection, then we could expect women of childbearing age to be susceptible to ZIKV after their primary infection (which might occur during the first epidemic wave).

## Transmission Seasonality

Seasonality is a common feature of acute infectious diseases [19–24], including *flaviviruses* like dengue virus (DENV), West Nile virus (WNV), yellow fever virus (YFV), and other arboviruses vectored by *Aedes aegypti* (i.e., chikungunya virus [CHIKV]) [25–29]. Although infectious diseases are seasonal, the timing of the high-transmission season can (1) vary among pathogens within a country and (2) vary among countries/regions for a given pathogen [30]. The drivers of DENV, WNV, YFV, and CHIKV seasonality are likely some combination of vector phenology, climate conditions, and additional host or environmental factors. In general, climatic, physiological, and behavioral factors that influence transmission seasonality include those that impact host and/or vector susceptibility to infection, host/vector infectiousness, virus viability, the transmission-relevant contact rate among hosts/vectors, the density of hosts, and vector abundance [19,24,30].

**Fig 1:**
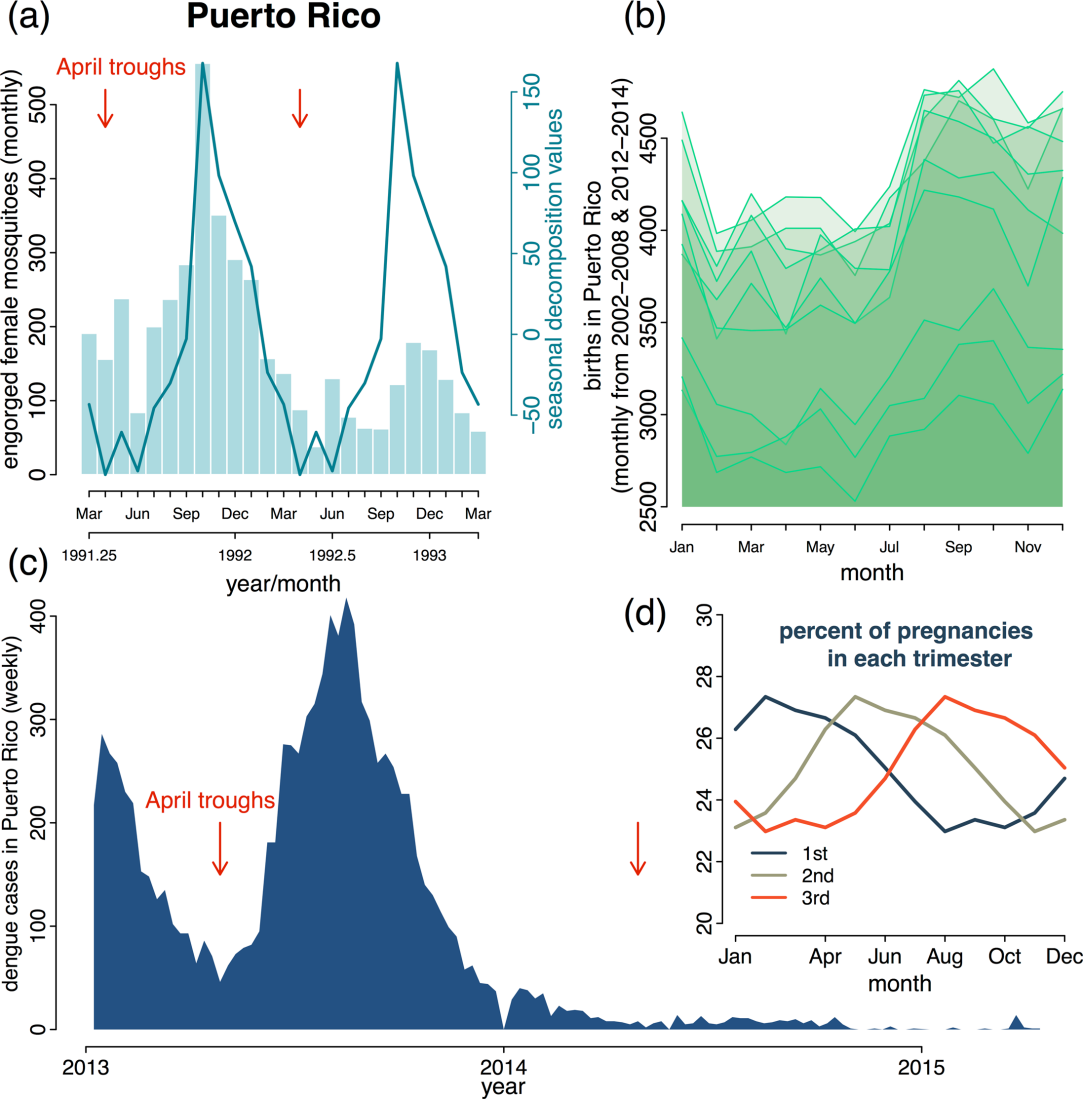
Seasonality of the ZIKV vector and birth seasonality. (a) Monthly abundance of trapped female mosquitoes engorged with blood-meal in Puerto Rico. Data from [36], available in S1 Data and S4 Data. Mosquitoes were collected from 20 houses in heavily urbanized areas around San Juan Metropolitan area using battery-powered aspirators. Seasonal time series decomposition was used to extract the seasonal component of the time series (solid line). Red arrows indicate the inferred seasonal trough of engorged female mosquitoes, which occurred around April each year. (b) Monthly births in Puerto Rico for the years 2002–2008 and 2012–2014. Data from [37], available in S2 Data and S4 Data. Time series from each year are stacked, with each line representing data from a single year. Births are seasonal around the world; the birth peak in Puerto Rico occurs around September each year. (c) Weekly reported DENV cases in Puerto Rico. Data are from [38], available in S3 Data and S4 Data. Red arrows indicate the inferred DENV transmission trough in April, which coincides with the trough in mosquitoes. (d) The seasonal distribution of pregnancies by trimester. The seasonal distribution of pregnancies by trimester was estimated based on the birth data from Puerto Rico. S4 Data is an R-programming package containing a function for calculating the seasonal distribution of pregnancies by trimester.

*Aedes aegypti* has seasonal variation in its ability to facilitate *flavivirus* transmission because its abundance and competence as a vector are affected by temperature and rainfall [31,32]. Using data from Puerto Rico—one of the US locations with ongoing ZIKV transmission—Fig 1A demonstrates the seasonal abundance of blood-fed female *Aedes aegypti*, which transmit ZIKV. *Aedes aegypti* seasonality affects seasonal transmission of DENV and CHIKV [31,33] and it is likely to impact seasonal ZIKV transmission. In regions with strong seasonal fluctuations in *Aedes aegypti*, seasonal changes in abundance and vector competence should be characterized and used to estimate the local timing of the high-transmission season.

A key reason for characterizing transmission seasonality and pinpointing the high transmission season is because its timing will affect the risk of microcephaly in birth cohorts. This is because births are seasonal across human populations, and there is a distinct birth pulse in most countries/regions that varies geographically in its seasonal timing [34,35]. Fig 1B shows the birth seasonality in Puerto Rico, with the birth peak from August–October. Due to birth seasonality, the percent of pregnancies experiencing a specific trimester is not evenly distributed throughout the year (Fig 1D). For any given country, the timing of the seasonal birth pulse relative to the ZIKV transmission season will therefore determine the fraction of pregnancies at risk for maternal infection and congenital ZIKV. For example, if a country has a birth pulse in which sensitive gestational periods coincide with the ZIKV season, more pregnancies in that country will be at risk than elsewhere. Fortunately, if access to contraceptives and family planning practices are proactively targeted for intervention, then the birth pulse could be intentionally shifted and amplified regionally to minimize the risk of intrauterine ZIKV infection for entire birth cohorts.

## Seasonally Timing Pregnancy

At this time, there are insufficient data to predict the seasonal timing and frequency (i.e., annual, biennial, triennial, etc.) at which ZIKV epidemics will occur. The ZIKV outbreak in Brazil peaked between July 12–18, 2015 [39], which is out-of-phase with DENV epidemics in Brazil, which consistently peak around March [40]. This suggests the seasonal timing of DENV epidemics might not be useful in predicting the timing of ZIKV. Importantly, however, the first epidemic wave of ZIKV may not reflect ZIKV’s future recurrent epidemic timing. The first wave of the epidemic may differ from future recurrent epidemics because (1) clinical recognition and reporting of cases may lag far behind pathogen introduction, (2) the first wave occurs in a fully susceptible population, which will alter the epidemic growth curve and the time until susceptible depletion, and (3) the timing of epidemic onset will be influenced by pathogen introduction as opposed to recurrent epidemics in locations with unbroken transmission chains, the onset of which is influenced by the build-up of the susceptible population and seasonal transmission [20].

**Fig 2:**
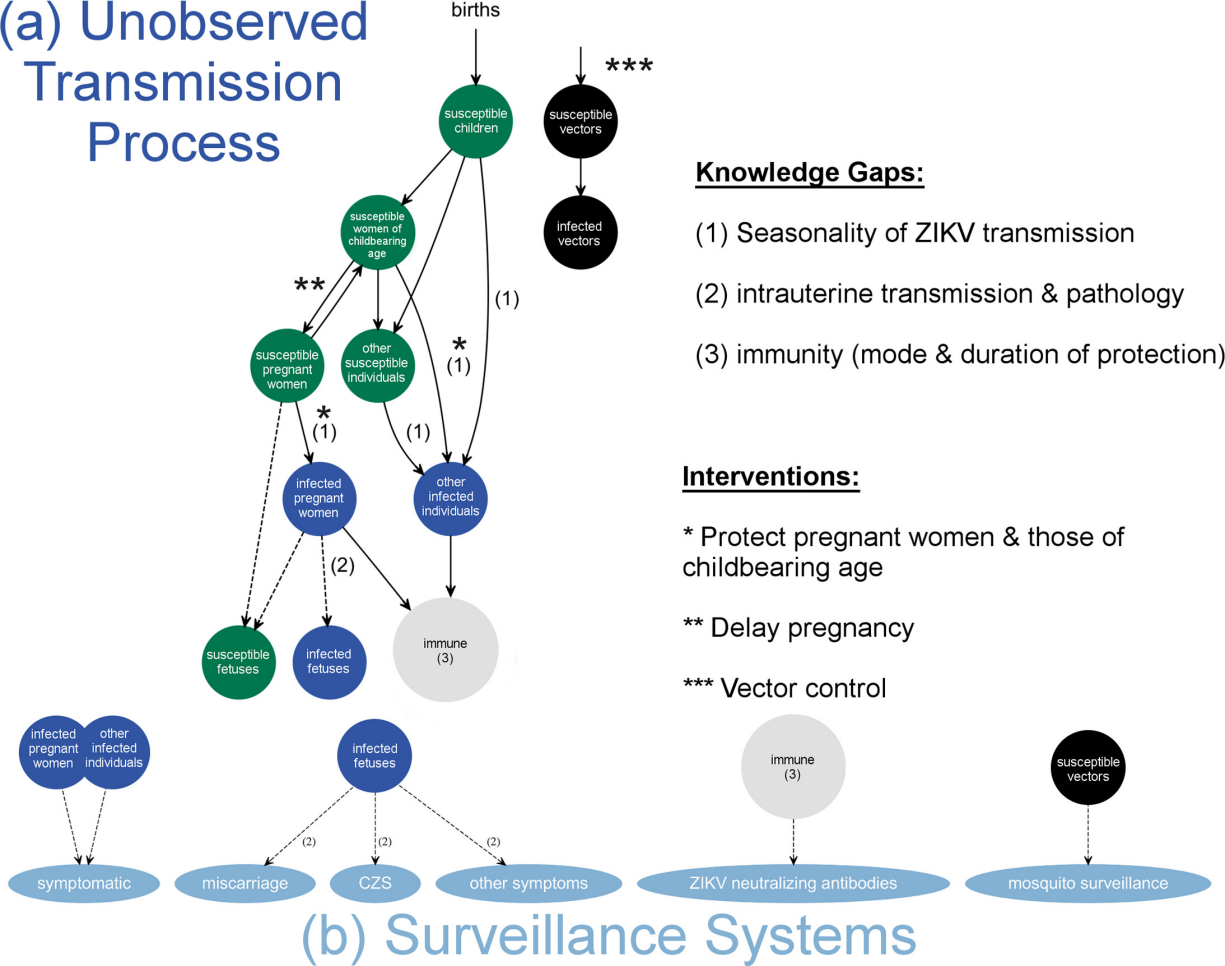
ZIKV transmission and pathology model schematic. Susceptible-Infected-Recovered model of ZIKV transmission. (a) Transmission model. Births enter the population seasonally, as illustrated by Fig 1B. Susceptible individuals are categorized as infants and children (“susceptible children”), adult males and women past reproductive age (“other susceptible individuals”), women of childbearing age, and pregnant women. There are three infected classes. The general infected class (“other infected individuals”) includes infants, children, adult males, and non-pregnant women. The infected class relevant to congenital ZIKV tracks pregnant women, who can transmit trans-placentally to infect their fetus. The recovered class (“immune”) contains individuals who have recovered from infection and are immune from infection, but it is unknown how long immunity lasts and the nature of repeat infection, indicated by knowledge gap 3. The transmission process is unobserved because transmission events are not captured in surveillance. Interventions are indicated. (b) Surveillance model. Symptomatic ZIKV infections and past infections are observable through surveillance systems. The model assumes a subset of infected individuals will have symptoms and the infection of a fetus can result in miscarriage, congenital Zika syndrome (CZS), or other forms of disease, the risk of which remains unknown (knowledge gap 2). Immunity in the host population can be observed through serology surveys, and mosquito abundance (“susceptible vectors” and “infected vectors”) can be measured based on vector surveillance. Vector abundance is seasonal as illustrated by Fig 1A and this likely affects seasonal transmission, indicated by knowledge gap 1.

As ZIKV incidence data become available, the annual transmission “high-season” and “low-season” should be characterized so pregnancy may be planned such that sensitive periods of gestation are aligned with the low-season window of opportunity. After the initial wave of the epidemic, ZIKV transmission models can be fitted and transmission parameters estimated using time series data from ZIKV surveillance. Fig 2 provides a potential ZIKV Susceptible-Infected-Recovered transmission model developed with a focus on the demography relevant to congenital ZIKV. To estimate seasonal transmission parameters, this model would require extensive time series data on reported ZIKV cases either weekly or monthly. To overcome data limitations, data from other ZIKV surveillance systems could be used in parallel to parameterize such a model. Surveillance data that could be used to study ZIKV transmission and pathology include reported cases, registries of miscarriage and CZS, ZIKV serology data, and mosquito surveillance data. Models with similar levels of complexity in transmission, pathology, and demography have been parameterized for poliovirus and measles [35,41]; see [42] for statistical inference methods. By combining transmission models with reported ZIKV cases, data on vector abundance, and other covariates that could influence transmission (e.g., temperature, humidity, and human movement), the underlying mechanistic drivers of ZIKV transmission seasonality could be revealed (knowledge gap 1). Assuming vector abundance is an important driver of ZIKV seasonal transmission, based on the *Aedes aegypti* data from Puerto Rico, the high-transmission season in Puerto Rico would occur between October–December and the trough would be April–June (Fig 1A). The impact of vector abundance on *flavivirus* transmission is indicated by the 2013 DENV epidemic in Puerto Rico, which had a trough in April (Fig 1C), as would be predicted based on vector seasonality. Importantly, the high and low transmission seasons are tied to local climate conditions and will therefore be region-specific.

With knowledge of regional transmission seasonality, initiating pregnancy during the seasonal window of opportunity for conception would minimize risk of maternal infection and subsequent damage to the fetus. Birth defects resulting from in utero infection with CMV, herpes simplex, and rubella virus are reported to be highest when maternal infection occurs within the first 20 weeks of gestation [43–45]. Miscarriages of known ZIKV-positive fetuses have been reported at 11 and 13 weeks gestation [8]. Preliminary data suggest miscarriage and CZS are most likely when maternal infection occurs during the first or second trimester [5,6,46,47], but fetal abnormalities have been found in women infected with ZIKV during weeks 8–35 of gestation [2], indicating all three trimesters are vulnerable to some extent. The critical window of susceptibility for ZIKV-induced miscarriage and CZS needs to be identified and taken into account when determining the seasonal window of opportunity for conception. Identifying the period of susceptibility for the fetus and using planned seasonal conception to redistribute births—i.e., to take advantage of the transmission low season and ensure sensitive gestation occurs during the ZIKV low season—would reduce risk to the fetus by minimizing maternal exposure. Based on the size of the 2014 birth cohort in Puerto Rico, redistributing births even by a small amount, for example with as little as 3% fewer births experiencing a susceptible trimester during the high transmission season, would translate to reducing risk for approximately 1,000 births annually. In a large country like Brazil, which had a birth cohort of approximately 3 million in 2015 [48], planned seasonal conception for 3% of births could reduce risk in >88,000 pregnancies. The absolute reduction in risk, however, is unknown, as it will depend on the incidence of ZIKV infection in the population and the subsequent risk of trans-placental transmission and fetal abnormalities.

**Fig 3:**
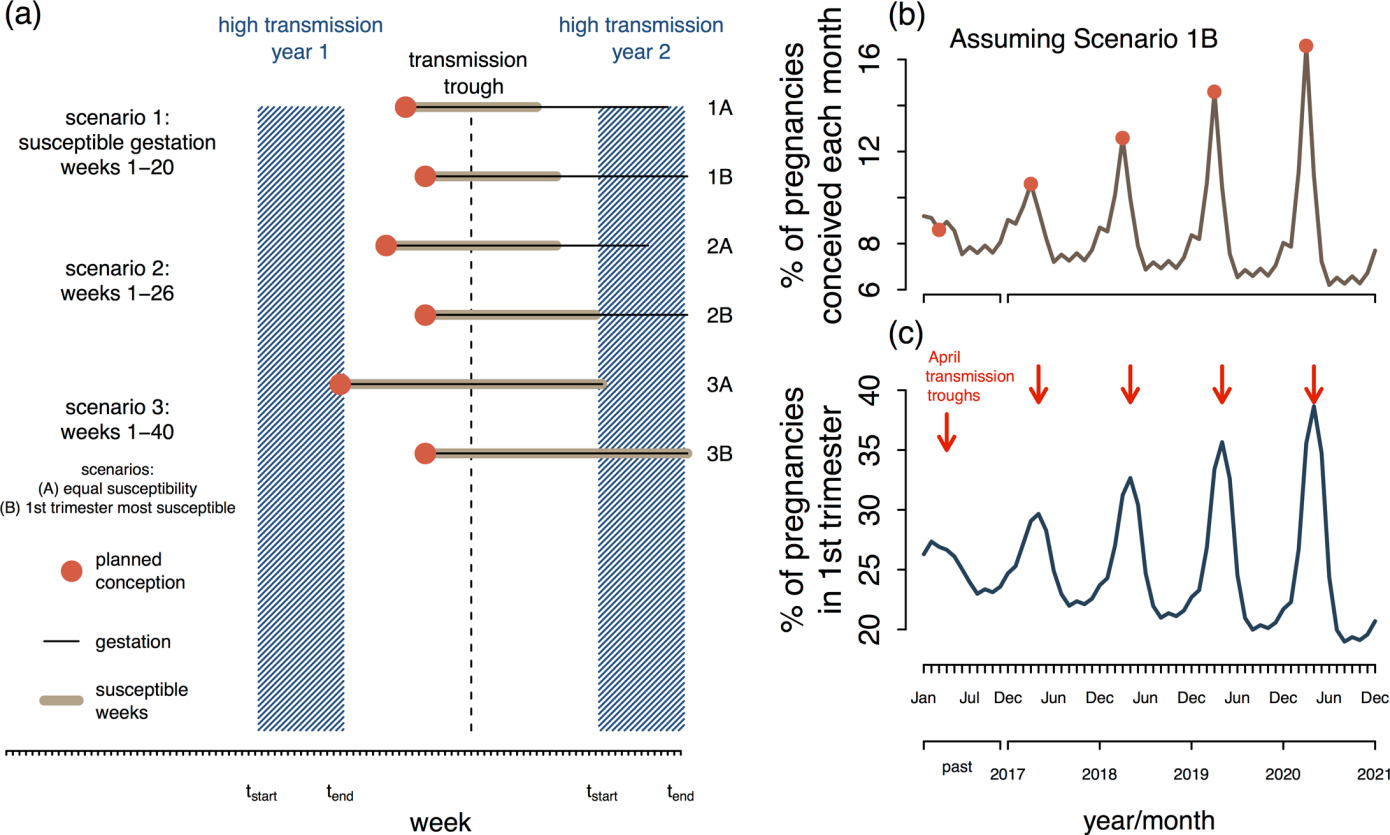
Planning pregnancy to take advantage of the low ZIKV transmission season. (a) Window of opportunity for conception based on the region-specific transmission season and the susceptible period of gestation. The high-transmission season is marked in blue, and the transmission trough is indicated by the dashed line. The ZIKV-susceptible gestation period is unknown, but preliminary data suggest that all three trimesters are susceptible; however, the first two trimesters are particularly vulnerable. The six scenarios depict the window of opportunity for conception under different susceptible gestation periods. Scenario 1 is susceptibility during gestation weeks 1–20, similar to other congenital infections. Scenario 2 is susceptibility during weeks 1–26, i.e., the first and second trimester. Scenario 3 is susceptibility during weeks 1–40, all three trimesters. The A and B variants of each scenario indicate whether there is uniform susceptibility across the susceptible gestation period or if the first trimester is particularly vulnerable. Policy could encourage planned conception such that the susceptible gestation period is aligned with the transmission trough. This policy would minimize the risk of maternal exposure when the fetus is most vulnerable. The window of opportunity for conception varies depending on when (in the calendar year) the high-transmission season occurs. (b) Theoretical trajectory of conception in Puerto Rico if conception were seasonally planned. The percent indicates the monthly value based on pregnancies initiated that year. The transmission trough was assumed to be in April (based on the data in Fig 1A and 1C). Gestation weeks 1–20 were assumed to be susceptible, with the first trimester being particularly vulnerable and given highest priority for protection. Conception was therefore encouraged in March, which had the effect of shifting and amplifying the birth pulse. The projection assumes that, each year, planned conception results in 3% of annual births that would have occurred in Jan., May., Jun., Jul., Aug., Sep., Oct., Nov., or Dec. being redistributed to Feb (0.5%), Mar (2%), and Apr (0.5%). (c) The seasonal distribution of pregnancies in the first trimester based on the conception trajectory in b. S5 Data provides R-code used to produce Fig 3B and 3C. S6 Data and S7 Data contain the time series plotted in Fig 3B and 3C, respectively.

Fig 3A shows the window of opportunity for conception when the high transmission season lasts 13 weeks and the fetus is susceptible during various periods of gestation. The window of opportunity depends on three key factors: (1) the timing of the transmission trough (i.e., the week(s) or month of the year when transmission is at a minimum), (2) the susceptible period of gestation, and (3) whether the severity of congenital ZIKV infection varies during the susceptible period of gestation. For example, it may be that the first two trimesters are susceptible to fetal abnormalities, but the first trimester is the most vulnerable. Knowing the distribution of susceptibility throughout the gestational weeks would impact the timing of planned conception. Assuming the period of susceptibility spans gestation weeks 1–20, with the first trimester being highly susceptible and therefore given high priority for protection, Fig 3B and 3C show how the seasonal distribution of conception could be shifted and amplified to reduce ZIKV risk in Puerto Rico. In general, although tailoring conception seasonally will not alleviate risk of maternal exposure to ZIKV, it could reduce risk and provide an option for women as they wait for a ZIKV vaccine and/or clinical interventions. Planned seasonal conception would be an effective low-cost means of empowering women to protect themselves and their children.

The feasibility and implementation of this strategy would require collaboration among vector ecologists, epidemiologists, and social scientists. In order to seasonally time pregnancy,

1. each country will need to identify their region-specific high and low ZIKV transmission season,
2. women and health care providers will need to be educated about seasonal conception, and
3. women will need access to contraception.

A key unknown is the susceptible period of gestation; when this period is determined, then seasonally planning pregnancy could be integrated into the growing portfolio of ZIKV interventions. The feasibility and acceptability of planning conception seasonally will need to be addressed regionally with careful consideration of women’s reproductive rights and personal values. An R-package including data used in this manuscript and a conception planning calendar is provided in S4 Data. The conception planner requires user-defined (1) timing of the transmission trough, (2) susceptible weeks of gestation, and (3) a statement of whether the first trimester is particularly vulnerable. To increase the effectiveness of seasonally planning conception, vector control campaigns could be used to restrict the mosquito season, minimize the duration of the high-transmission season, and expand the window of opportunity for “safe gestation.” The integration of epidemiology and family planning can be an effective tool for seasonally timing conception to reduce women’s risk of ZIKV infection during pregnancy.

## Supporting Information

S1 Data Mosquito abundance. Monthly abundance of trapped female mosquitoes engorged with blood-meal in Puerto Rico. Data from [36].
(CSV)

S2 Data Births. Monthly births in Puerto Rico for the years 2002–2008 and 2012–2014. Data from [37].
(CSV)

S3 Data Dengue cases. Weekly reported DENV cases in Puerto Rico. Data are from [38].
(CSV)

S4 Data R-package: ZIKV. R-package containing data and functions associated with this manuscript.
(GZ) Click here for additional data file.

S5 Data R-code. R-code used to produce Fig 3B and 3C.
(R) Click here for additional data file.

S6 Data Fig 3B data. Time series produced using S5 Data and plotted in Fig 3B.
(CSV)

S7 Data Fig 3C data. Time series produced using S5 Data and plotted in Fig 3C.
(CSV)

S8 Data R-package: ZIKV manual. PDF manual for the R-package: ZIKV provided as S4 Data.
(PDF)

## Abbreviations

CDC: Centers for Disease Control and Prevention
CHIKV: chikungunya virus
CNS: Central Nervous System
CZS: congenital Zika syndrome
DENV: dengue virus
WHO: World Health Organization
WNV: West Nile Virus
YFV: Yellow fever virus
ZIKV: Zika virus

## References

[1] Fauci AS, Morens DM. ZIka Virus in the Americas—Yet Another Arbovirus Threat. The New England Journal of Medicine. 2016;374:601–604. 10.1056/NEJMp1002530 26761185

[2] Brasil P, Pereira JP Jr, Raja Gabaglia C, Damasceno L, Wakimoto M, Ribeiro Nogueira RM, et al Zika Virus Infection in Pregnant Women in Rio de Janeiro—Preliminary Report. New England Journal of Medicine. 2016; 10.1056/NEJMoa1602412 PMC5323261 26943629

[3] Hazin A, Poretti A, Di Cavalcanti Souza Cruz D, Tenorio M, van der Linden A, Pena L, et al Computed Tomographic Findings in Microcephaly Associated with Zika Virus. The New England Journal of Medicine. 2016; p. 1–3. 10.1056/NEJMc1603617 27050112

[4] World Health Organization. Zika: Strategic Response Framework & Joint Operations Plan January-June 2016; 2016. Feb 2016.

[5] Rasmussen SA, Jamieson DJ, Honein MA, Petersen LR. Zika Virus and Birth Defects—Reviewing the Evidence for Causality. The New England Journal of Medicine. 2016; p. 1–7. 10.1056/NEJMsr1604338 27074377

[6] Besnard M, Eyrolle-Guignot D, Guillemette-Artur P, Lastere S, Bost-Bezeaud F, Marcelis L, et al Congenital Cerebral Malformations and Dysfunction in Fetuses and Newborns Following the 2013 to 2014 Zika Virus Epidemic in French Polynesia. Euro Surveillance. 2016;21(13):30181 10.2807/1560-7917.ES.2016.21.13.30181 27063794

[7] Mlakar J, Korva M, Tul N, Popović M, Poljšak-Prijatelj M, Mraz J, et al Zika Virus Associated with Microcephaly. New England Journal of Medicine. 2016; p. 160210140106006 10.1056/NEJMoa1600651 26862926

[8] Martines RB, Bhatnagar J, Keating MK, Silva-flannery L, Gary J, Goldsmith C, et al Evidence of Zika Virus Infection in Brain and Placental Tissues from Two Congenitally Infected Newborns and Two Fetal Losses—Brazil, 2015. Morbidity and Mortality Weekly Report. 2016;65(6):159–160. 10.15585/mmwr.mm6506e1 26890059

[9] Miner J, Cao B, Govero J, Smith A, Fernandez E, Cabrera O, et al Zika Virus Infection during Pregnancy in Mice Causes Placental Damage and Fetal Demise. Cell. 2016; p. 1–11. 10.1016/j.cell.2016.05.008 PMC4874881 27180225

[10] Cugola FR, Fernandes IR, Russo FB, Freitas BC, Dias JLM, Guimarães KP, et al The Brazilian Zika Virus Strain Causes Birth Defects in Experimental Models. Nature. 2016; p. 1–15. 10.1038/nature18296 PMC4902174 27279226

[11] Gareez P, Correia Loiola E, Madeiro da Costa R, Higa LM, Trindade P, Delvecchio R, et al Zika Virus Impairs Growth in Human Neurospheres and Brain Organoids. Science. 2016;352(6287):816–818. 10.1086/429930 27064148

[12] Obama B. Letter from the President—Zika Virus. The White House Office of the Press Secretary. Feb 22, 2016.

[13] Frieden T. Preparing and Responding to Zika Virus. In: Zika Action Plan Summit. April; 2016.

[14] World Health Organization. WHO Zika Q&A; 2016. http://www.who.int/features/qa/zika/en/.

[15] Centers for Disease Control and Prevention, National Center for Emerging and Zoonotic Infectious Diseases, Division of Vector-Borne Diseases. Zika Virus: Women and Their Partners Who are Thinking about Pregnancy. http://www.cdc.gov/zika/pregnancy/thinking-about-pregnancy.html.

[16] McNeil DG Jr. Health Officials Split Over Advice on Pregnancy in Zika Areas. The New York Times Apr 14, 2016 http://nyti.ms/1SF3uQt.

[17] Tavernise S. C.D.C. Offers Guidelines for Delaying Pregnancy After Zika Exposure. The New York Times Mar 25, 2016 http://nyti.ms/1RE96A8.

[18] McNeil DG Jr. Growing Support Among Experts for Zika Advice to Delay Pregnancy. The New York Times 2 5, 2016 http://nyti.ms/1UQdQjl.

[19] Altizer S, Dobson A, Hosseini P, Hudson P, Pascual M, Rohani P. Seasonality and the Dynamics of Infectious Diseases. Ecology Letters. 2006;9(4):467–484. 10.1111/j.1461-0248.2005.00879.x 16623732

[20] Grassly NC, Fraser C. Seasonal Infectious Disease Epidemiology. Proceedings of the Royal Society B: Biological Sciences. 2006;273:2541–2550. 10.1098/rspb.2006.3604 16959647 PMC1634916

[21] Dowell SF. Seasonality—Still Confusing. Epidemiology and Infection. 2012;140:87–90. 10.1017/S0950268811001695 21906417

[22] Fisman DN. Seasonality of Infectious Diseases. Annual Review of Public Health. 2007;28:127–143. 10.1146/annurev.publhealth.28.021406.144128 17222079

[23] Dowell SF. Seasonal Variation in Host Susceptibility and Cycles of Certain Infectious Diseases. Emerging Infectious Diseases. 2001;7(3):369–374. 10.3201/eid0703.010301 11384511 PMC2631809

[24] Martinez-Bakker M, Helm B. The Influence of Biological Rhythms on Host-Parasite Interactions. Trends in Ecology and Evolution. 2015;30(6):314–326. 10.1016/j.tree.2015.03.012 25907430

[25] Brady OJ, Golding N, Pigott DM, Kraemer MUG, Messina JP, Reiner RC, et al Global Temperature Constraints on *Aedes aegypti* and *Ae*. *albopictus* Persistence and Competence for Dengue Virus Transmission. Parasites & Vectors. 2014;7(1):338 10.1186/1756-3305-7-338 25052008 PMC4148136

[26] Hayes EB, Komar N, Nasci RS, Montgomery SP, O’Leary DR, Campbell GL. Epidemiology and Transmission Dynamics of West Nile Virus Disease. Emerging Infectious Diseases. 2005;11(8):1167–1173. 10.3201/eid1108.050289a 16102302 PMC3320478

[27] Romano APM, Costa ZGA, Ramos DG, Andrade MA, de Jayme VS, de Almeida MAB, et al Yellow Fever Outbreaks in Unvaccinated Populations, Brazil, 2008–2009. PLoS Negl Trop Dis. 2014;8(3):18–21. 10.1371/journal.pntd.0002740 PMC3953027 24625634

[28] Ruiz-Moreno D, Vargas IS, Olson KE, Harrington LC. Modeling Dynamic Introduction of Chikungunya Virus in the United States. PLoS Negl Trop Dis. 2012;6(11):1–8. 10.1371/journal.pntd.0001918 PMC3510155 23209859

[29] Johansson MA, Powers AM, Pesik N, Cohen NJ, Erin Staples J. Nowcasting the Spread of Chikungunya Virus in the Americas. PLoS ONE. 2014;9(8). 10.1371/journal.pone.0104915 PMC4128737 25111394

[30] Stevenson TJ, Visser ME, Arnold W, Barrett P, Biello S, Dawson A, et al Disrupted Seasonal Biology Impacts Health, Food Security and Ecosystems. Proceedings of the Royal Society B: Biological Sciences. 2015;282:20151453 10.1098/rspb.2015.1453 26468242 PMC4633868

[31] Lambrechts L, Paaijmans KP, Fansiri T, Carrington LB, Kramer LD, Thomas MB, et al Impact of Daily Temperature Fluctuations on Dengue Virus Transmission by *Aedes aegypti*. Proceedings of the National Academy of Sciences of the United States of America. 2011;108(18):1–6. 10.1073/pnas.1101377108/-/DCSupplemental.www.pnas.org/cgi/doi/10.1073/pnas.1101377108 PMC3088608 21502510

[32] Soper FL. Dynamics of *Aedes aegypti* Distribution and Density. Bulletin of the World Health Organization. 1967;36:536–538. 5299446 PMC2476418

[33] Pialoux G, Bernard-Alex G, Jaureguiberry S, Strobel M. Chikungunya, An Epidemic Arbovirosis. Lancet Infectious Diseases. 2007;7(5):319–327. 10.1016/S1473-3099(07)70107-X 17448935

[34] Roenneberg T, Aschof J. Annual Rhythm of Human Reproduction: I. Biology, Sociology, or Both? Journal of Biological Rhythms. 1990;5(3):195–216. 10.1177/074873049000500303 2133132

[35] Martinez-Bakker M, Bakker K, King AA, Rohani P. Human Birth Seasonality: Latitudinal Gradient and Interplay with Childhood Disease Dynamics. Proceedings of the Royal Society B: Biological Sciences. 2014;281(1783). 10.1098/rspb.2013.2438 PMC3996592 24695423

[36] Scott TW, Morrison AC, Lorenz LH, Clark GG, Strickman D, Kittayapong P, et al Longitudinal Studies of *Aedes aegypti* (Diptera: Culicidae) in Thailand and Puerto Rico: Population Dynamics. Journal of Medical Entomology. 2000;37(1):77–88. 15218910 10.1603/0022-2585-37.1.77

[37] United Nations Statistics Division: Demographics Statistics. Live births by month of birth. 2016. http://data.un.org/.

[38] Centers for Disease Control and Prevention. National Notifiable Disease Surveillance System (NNDSS). Morbidity and Mortality Weekly Report. Data available at https://data.cdc.gov/. Downloaded May 2 2016.

[39] Faria NR, do Socorro da Silva Azevedo, Kraemer MUG, Souza R, Cunha MS, Hill SC, et al Zika Virus in the Americas: Early Epidemiological and Genetic Findings. Science. 2016;352(April):345–349. 10.1126/science.aaf5036 27013429 PMC4918795

[40] San Martin JL, Brathwaite O, Zambrano B, Solorzano JO, Bouckenooghe A, Dayan GH, et al The Epidemiology of Dengue in the Americas over the Last Three Decades: A worrisome Reality. American Journal of Tropical Medicine and Hygiene. 2010;82(1):128–135. 10.4269/ajtmh.2010.09–0346 20065008 PMC2803522

[41] Martinez-Bakker M, King AA, Rohani P. Unraveling the Transmission Ecology of Polio. PLoS Biol. 2015;13(6):e1002172 10.1371/journal.pbio.1002172 26090784 PMC4474890

[42] King AA, Nguyen D, Ionides E. Statistical Inference for Partially Observed Markov Processes via the R Package pomp. Journal of Statistical Software. 2015;(in press).

[43] Pass RF, Fowler KB, Boppana SB, Britt WJ, Stagno S. Congenital Cytomegalovirus Infection Following First Trimester Maternal Infection: Symptoms at Birth and Outcome. Journal of Clinical Virology. 2006;35(2):216–220. 10.1016/j.jcv.2005.09.015 16368262

[44] Sever J, White L. Intrauterine Viral Infections. Annual Review of Medicine. 1968;19(1):471–486. 10.1146/annurev.me.19.020168.002351 4172728

[45] Sauerbrei A, Wutzler P. Herpes Simplex and Varicella-Zoster Virus Infections During Pregnancy: Current Concepts of Prevention, Diagnosis and Therapy. Part 1: Herpes Simplex Virus Infections. Medical Microbiology and Immunology. 2007;196(2):89–94. 10.1007/s00430-006-0031-0 17165093

[46] Cauchemez S, Besnard M, Bompard P, Dub T, Guillemette-Artur P, Eyrolle-Guignot D, et al Association between Zika virus and Microcephaly in French Polynesia, 2013–15: A Retrospective Study. The Lancet. 2016;6736(16):1–8. 10.1016/S0140-6736(16)00651-6 PMC4909533 26993883

[47] de Oliveira WK, Cortez-escalante J, Tenório W, Holanda G, Madeleine G. Increase in Reported Prevalence of Microcephaly in Infants Born to Women Living in Areas with Confirmed Zika Virus Transmission During the First Trimester of Pregnancy—Brazil, 2015. MMWR Morbidity and Mortality Weekly Report. 2016;65(9):242–247. 10.15585/mmwr.mm6509e2 26963593

[48] US Central Intelligence Agency. The World Factbook 2016; 2016. https://www.cia.gov/library/publications/the-world-factbook/index.html.

