# Supplementary material for: Preventing Zika Virus Infection during Pregnancy Using a Seasonal Window of Opportunity for Conception
Source: PLoS Biol. 2016 Jul 28;14(7):e1002520. doi: 10.1371/journal.pbio.1002520 (PMC4965063; doi:10.1371/journal.pbio.1002520)
Supplement: S8 Data — PDF manual for the R-package: ZIKV provided as S4 Data. (PDF) [file pbio.1002520.s008.pdf]

Package ‘ZIKV’

June 13, 2016

Title R Package associated with the manuscript ``Preventing Zika Virus Infection during Pregnancy using a Seasonal Window of Opportunity for Conception''

Version 1.1

Date 2016-05-13

Description This R package includes data used in the manuscript ``Preventing Zika Virus Infection during Pregnancy by Timing Conception Seasonally''. It also contains a function to generate region-specific conception planning calendars. The conception planner function requires user defined (1) timing of the ZIKV transmission trough, (2) susceptible weeks of gestation, and (3) a statement of whether the first trimester is particularly vulnerable to congenital Zika.

Depends R (>= 3.2.4), grDevices (>= 3.2.4)

License GPL (>= 2)

LazyData true

NeedsCompilation no

Author Micaela Martinez-Bakker [aut, cre]

Maintainer Micaela Martinez-Bakker <micaela.martinezbakker@gmail.com>

R topics documented:

births 1
conception.planner 2
dengue 3
mosquitoes 3
planned.conception 4
trimester.monthly.distribution 5

Index **6**

`births` *Monthly births in Puerto Rico.*

Description

Monthly number of births in Puerto Rico.

Usage

```
births
```

Format

A data frame.

Source

Data obtained from:

United Nations Statistics Division: Demographics Statistics. Live births by month of birth. 2016. Available from: http://data.un.org/.

References

United Nations Statistics Division: Demographics Statistics. Live births by month of birth. 2016. Available from: http://data.un.org/.

---

conception.planner *Plot Conception Planning Calendar.*

Description

Function for creating and plotting a conception calendar to align sensitive periods of gestation with the low-transmission season for ZIKV. It returns a data frame showing which week of the year each gestation week should be aligned with. There are assumptions regarding the weeks in each trimester. We assume trimester 1 is weeks 1:13, trimester 2 is weeks 14:26, and trimester 3 is weeks 27:40.

Usage

```
conception.planner(transmission.trough.week = NA, start.susceptible = 1,
  end.susceptible = 20, first.trimester.priority = TRUE,
  directory = getwd(), figure.name = "conception_planner.pdf")
```

Arguments

`transmission.trough.week`
: the calendar week (1-52) of the year with the lowest ZIKV transmission.

`start.susceptible`
: the week of gestation (1-40) when the fetus is first susceptible to congenital Zika.

`end.susceptible`
: the last week of gestation (1-40) that the fetus is susceptible to congenital Zika.

`first.trimester.priority`
: TRUE/FALSE, if TRUE the first trimester is most vulnerable to congenital Zika and the first trimester will be aligned with the transmisison trough.

`directory`
: the directory where the pdf should be saved

`figure.name`
: the name of the pdf file, ending in ".pdf"

Examples

```
conception.plan<- conception.planner(
transmission.trough.week=20,
start.susceptible= 1,
end.susceptible= 20,
first.trimester.priority=TRUE,
directory=getwd(),
figure.name='conception_planner.pdf')
```

`dengue` *Dengue cases in Puerto Rico.*

Description

Weekly Dengue virus cases reported in Puerto Rico.

Usage

```
dengue
```

Format

A data frame.

Source

Data were obtained from: Centers for Disease Control and Prevention. National Notifiable Disease Surveillance System (NNDSS). Morbidity and Mortality Weekly Report. Data available at https://data.cdc.gov/

References

Data were obtained from: Centers for Disease Control and Prevention. National Notifiable Disease Surveillance System (NNDSS). Morbidity and Mortality Weekly Report. Data available at https://data.cdc.gov/

`mosquitoes` *Seasonal abundance of engorged female Aedes aegypti mosquitoes.*

Description

Monthly number of engorged female Aedes aegypti mosquitoes trapped in 20 houses in urban areas of Puerto Rico.

Usage

```
mosquitoes
```

Format

A data frame.

Source

The data were digitized from:

Scott TW, Morrison AC, Lorenz LH, Clark GG, Strickman D, Kittayapong P, et al. Longitudinal Studies of Aedes aegypti (Diptera: Culicidae) in Thailand and Puerto Rico: Population Dynamics. Journal of Medical Entomology. 2000;37(1):77–88.

References

Scott TW, Morrison AC, Lorenz LH, Clark GG, Strickman D, Kittayapong P, et al. Longitudinal Studies of Aedes aegypti (Diptera: Culicidae) in Thailand and Puerto Rico: Population Dynamics. Journal of Medical Entomology. 2000;37(1):77–88.

---

planned.conception *Calculate Recommended Timing of Conception.*

Description

Function for creating a conception calendar to align sensitive periods of gestation with the low-transmission season for ZIKV. It returns a data frame showing which week of the year each gestation week should be aligned with. There are assumption regarding the weeks in each trimester. We assume trimester 1 is weeks 1:13, trimester 2 is weeks 14:26, and trimester 3 is weeks 27:40.

Usage

```
planned.conception(transmission.trough.week = NA,
  susceptible.gestation.start.week = 1, susceptible.gestation.end.week = 20,
  first.trimester.priority = TRUE)
```

Arguments

`transmission.trough.week`
: the calendar week (1-52) of the year with the lowest ZIKV transmission.

`susceptible.gestation.start.week`
: the week of gestation (1-40) when the fetus is first susceptible to congenital Zika.

`susceptible.gestation.end.week`
: the last week of gestation (1-40) that the fetus is susceptible to congenital Zika.

`first.trimester.priority`
: TRUE/FALSE, if TRUE the first trimester is most vulnerable to congenital Zika and the first trimester will be aligned with the transmisison trough.

Examples

```
conception.plan<- planned.conception(
transmission.trough.week=20,
susceptible.gestation.start.week=1,
susceptible.gestation.end.week=20,
first.trimester.priority=TRUE)
```

`trimester.monthly.distribution` *Calculate Seasonal Distribution of Conception and Gestation.*

Description

Function for creating the seasonal distribution of conception and pregancies in the 1st, 2nd, and 3rd trimester, based on monthly birth data. The function a returns data frame containing the percent and number of births conceived each month, and the percent and number of the pregnancies in each trimester (monthly). It assumes 9 months of gestation, and months 1-3 are trimester 1, 4-6 are trimester 2, and 7-9 are trimester 3.

Usage

```
trimester.monthly.distribution(data)
```

Arguments

`data`	data must be a data frame with “year”, “month”, and “births” as column headers; month must be the full name of the month January, February, etc.

Examples

```
seasonal.distribution<- trimester.monthly.distribution(data=births)
```

Index

∗Topic **datasets**
- births, 1
- dengue, 3
- mosquitoes, 3

births, 1

conception.planner, 2

dengue, 3

mosquitoes, 3

planned.conception, 4

trimester.monthly.distribution, 5
